# The effectiveness of simple psychological and exercise interventions for high prevalence mental health problems in young people: a factorial randomised controlled trial

**DOI:** 10.1186/1745-6215-12-76

**Published:** 2011-03-13

**Authors:** Alexandra G Parker, Sarah E Hetrick, Anthony F Jorm, Alison R Yung, Patrick D McGorry, Andrew Mackinnon, Bridget Moller, Rosemary Purcell

**Affiliations:** 1 Orygen Youth Health Research Centre, Centre for Youth Mental Health, University of Melbourne, Locked Bag 10, Parkville, Victoria 3052, Australia

## Abstract

**Background:**

The prevalence of mental illness in young people is the highest of any age group, with the onset of depression, anxiety and substance use peaking between 18 and 24 years. Effective treatments that target sub-threshold or mild to moderate levels of disorder in young people are required to reduce the risk of persistence and recurrence. The aims of this study are to evaluate whether treatments that are less intensive than cognitive-behaviour therapy, such as problem solving therapy and exercise treatments, are acceptable and effective in managing depression and anxiety symptoms in young people and to identify possible attributes in those who are likely to respond to these treatments.

**Methods/design:**

This is a factorial randomised controlled trial conducted at a large, metropolitan youth mental health service. Participants are young help-seekers aged 15-25 years with sub-threshold or mild to moderate levels of depression and anxiety (with or without comorbid substance use). The interventions comprise 4 treatment combinations delivered by psychologists over 6 sessions on a weekly basis: a psychological intervention (problem solving therapy versus supportive counselling) and an exercise intervention (behavioural exercise versus psychoeducation). Structured assessments occur at baseline, mid-point, end-point (6 weeks) and at a 6- and 12-month follow-up. The primary outcomes are depression and anxiety symptoms as measured by the Beck Depression and Anxiety Inventories. Secondary outcomes include remission (defined as no longer meeting the diagnostic criteria for a disorder if threshold level was reached at baseline, or no longer scoring in the clinical range on scale scores if sub-threshold at baseline), substance use, and functioning.

**Discussion:**

The effectiveness of less complex psychological and exercise interventions in young help-seekers with sub-threshold or mild to moderate presentations of high prevalence disorders is yet to be explored. This study has been designed to examine the effectiveness of these interventions delivered alone, or in combination, in a youth-specific service. If effective, the interventions have the potential to prevent the progression of early symptoms and distress to later and potentially more serious stages of mental disorder and reduce the likelihood of ongoing problems associated with the risk of persistence and recurrence.

**Trial registration:**

Australian New Zealand Clinical Trials Registry ACTRN12608000550303

## Background

The prevalence of mental disorders in young people is the highest of any age group [1], with the onset of high prevalence disorders such as depression, anxiety and substance use peaking within the age range of 18 to 24 years [2]. There are many negative consequences, both immediate and long-term, associated with mental disorders, including impairments in social functioning, poor education and employment attainment and achievement [3-5], and increased risks of self-harm and suicide [6]. These outcomes do not solely occur in people with full-threshold or severe forms of disorder; considerable impairment in functioning is associated with what are often referred to as 'sub-threshold' mental health problems, which are equally, if not more, prevalent [4,7,8].

Despite the prevalence and adverse outcomes of experiencing mental health problems during adolescence and young adulthood, young people are often reluctant to seek help for mental health problems [9] and are the least likely of all age groups to receive appropriate mental health care [1,10]. To better facilitate the engagement of young clients, services need to be youth-friendly, delivered in a low-stigma setting and offer accessible and acceptable interventions for young people [11,12] that are developmentally appropriate [13,14]. Effective treatments for this population that target early phases or sub-threshold levels of disorder have the potential to reduce the risk of persistence and recurrence [3,15,16], and impairments in social, educational and vocational functioning [3-5].

A potentially useful framework to guide treatment decision-making is the clinical staging model for psychiatric disorders, which proposes matching specific interventions to specific stages of illness [17-19]. This model is predicated on the notion that treatments delivered earlier in the course of illness development will be safer and more effective than those delivered later in the course of more established illness, with earlier treatment potentially preventing progression to more severe forms of disorder [17]. For example, milder, yet potentially serious disorders at a sub-threshold or early stage may respond to simple interventions such as psychosocial support, self-help strategies and psycho-education, reserving more intensive psychotherapy and/or pharmacotherapy for later or more severe stages of illness. However, the majority of studies that have contributed to the current evidence base of interventions for young people with depression and anxiety have included participants with full-threshold levels of disorders (e.g., see [20-22] for systematic reviews). It remains unknown whether less complex or intensive interventions, often used as control conditions in treatment studies of more severe disorders, would be feasible and effective treatments for those experiencing milder or sub-threshold forms of mental health problems.

The majority of psychological treatment trials for young people with depression and/or anxiety disorders have used cognitive-behaviour therapy (CBT), or adaptations of this therapy, as the intervention [20,21]. These trials have typically included participants with moderate to severe full-threshold disorders of prolonged duration, typified by the two major studies of psychological and pharmacological therapies for depression; the Treatment for Adolescent Depression (TADS; [23]) and the Adolescent Depression Antidepressant and Psychotherapy Trial (ADAPT; [24]). Brief forms of this therapy have rarely been offered, with 12 sessions as standard [20] and most trials have been based in specialist mental health settings, making it unclear whether the intervention would be feasible or acceptable for young people with mild to moderate or sub-threshold forms of disorders who present for treatment in primary or enhanced primary care settings.

The multiple strategies in the mode of CBT disseminated in the TADS study has been criticised as being too dense, with simpler models of CBT that focus on one or two core components, such as behaviour activation and problem solving, argued to be more beneficial [25]. Indeed, a recent meta-analysis of psychotherapy for depressed children and adolescents demonstrated that both cognitive (i.e., CBT) and 'non-cognitive' (for example, behavioural activation, family therapy, behavioural problem solving, group support and social skills training) strategies were equally effective in treating depression [21], suggesting that the specific targeting of cognitions might not be a necessary component of effective treatment in young people [26]. Further, it has been argued that young people appear to be more interested in treatment that offers them a chance to be listened to and to learn new skills rather than develop a deeper understanding of the psychological processes that contribute to their behaviour [27]. Combined, these findings suggest the importance of exploring alternative psychotherapies to CBT that may be more acceptable and effective in younger populations with mild to moderate or sub-threshold levels of disorder.

Given the concerns raised with the necessity of targeting cognitive distortions in young people and the difficulties in disseminating CBT, we were interested in exploring psychological interventions that were less complex and could be delivered in a shorter number of sessions. It was also of interest to explore interventions that have the potential to be delivered in primary care settings, which may be more appropriate settings for young people with sub-threshold or mild to moderate high prevalence disorders. Furthermore, an overarching criticism of interventions for young people with depression and anxiety symptoms is that health risk behaviours that co-occur with mental health problems tend to be overlooked [26]. These health risk behaviours include substance use, eating problems and low levels of regular physical activity. Interventions that can incorporate behavioural activation, such as problem solving therapy [28-30] and exercise interventions [31], are beginning to be explored as potentially effective interventions in reducing depression and anxiety symptoms in young people.

Problem solving therapy (PST) aims to assist a person in learning to cope more effectively with their current difficulties, as well as developing skills that can be used in other settings and times in their life [32]. PST aims to achieve this by systematically generating solutions to current problems and implementing a structured plan to resolve the difficulties, thereby introducing new behaviours and skills to effectively solve everyday problems [33]. The therapy focuses on how to implement changes in the 'here and now' rather than working through the meaning and impact of past experiences [33,34]. Given the relationship between stressful life events and the early onset of depression [35], PST has face validity as an intervention among young people. To date, its effectiveness has been evaluated in two studies in this age group. In 2008, Eskin and colleagues [29] randomly assigned 46 self-referred high school or university students (mean age 19 years) who met DSM-IV criteria [36] for major depressive disorder to either PST (n = 27) or a wait-list control group (n = 19). At the end of treatment, the 6 individual sessions of PST were more effective than a wait-list control in reducing depression symptoms and suicidal ideation, as well as increasing assertiveness and self-esteem in this sample. These changes were maintained at a 12-month follow-up assessment. Arguably, changes in these constructs can assist young people in negotiating and responding to future challenges through the development of relevant skills. Participants allocated to the PST intervention attended all 6 sessions of the treatment, indicating that it is an acceptable form of treatment for young people with depression and suicidal ideation. However, the use of a wait-list control group may be an inadequate comparison. It has been criticised because it only allows for the control of the passage of time, but not for other potential non-specific therapeutic factors, such as supportive contact [37].

In an earlier and smaller trial by Lerner and Clum (1990), group-based PST (n = 9) was compared with supportive psychotherapy (n = 9) in young people (mean age 19 years) who had suicidal ideation and self-reported depressive symptoms, recruited through the psychology department of a university [30]. The design had an appropriate condition to control for non-specific therapeutic effects and each intervention consisted of 10 group sessions over a period of 5-7 weeks. PST was more effective than supportive therapy in reducing depressive symptoms and hopelessness, but suicidal ideation decreased by a similar amount in both treatment groups. Combined, these studies provide a preliminary evidence-base for using PST as an intervention with younger populations, in particular young people with depressive symptoms. Although Lerner and Clum's study contained 10 sessions, compared with the 6 used by Eskin and colleagues, the duration of the interventions were shorter than the average duration of CBT (see [20]). This is consistent with a recent meta-analysis of psychotherapy for depression in young people showing no correlation between treatment duration and outcome, indicating that briefer treatments may have a similar effect to those of longer duration [21].

As mentioned above, health risk behaviours, including low levels of physical activity, tend to be overlooked in psychological interventions with young people, although both mental health and physical health benefits have been found [26]. Exercise interventions for those with psychological difficulties are proposed to improve mood by interrupting or distracting the person from dysfunctional or negative thoughts and by the release of endorphins via strenuous exercise [38]. Weekly monitoring of physical activity within psychotherapy sessions has been found to be effective in reducing depression and anxiety symptoms [39]. A key component of the success of exercise interventions appears to be the encouragement and assistance provided to participants to engage in regular physical activity, as well as benefits derived from assessment and routine monitoring of daily levels of exercise (e.g., see [40]).

Although based on a small number of trials of variable quality, a systematic review of exercise interventions in predominantly healthy children and young people found positive short-term effects on self-esteem, with no reported adverse effects [41]. A more recent systematic review of exercise in the prevention and treatment of depression and anxiety in children and young people [31] found a small effect in favour of exercise, however, again, the number of included trials was small and their quality variable. Whether the exercise interventions were of high or low intensity made little difference to the outcomes. The evidence base for the effectiveness of exercise interventions in children and young people who are experiencing mental health problems is scarce as the majority of trials have been conducted in healthy populations. Further high-quality studies are required to evaluate whether exercise is an effective treatment for depression and anxiety in young people, particularly those with emerging symptomatology.

As noted above, it is essential to include an active control group when designing a treatment trial in order to control for placebo or expectancy effects, or for the non-specific effects of a therapeutic relationship [21]. We believe this is also warranted when conducting studies in clinical help-seeking populations. Control groups with comparable intensity and duration of exposure to the active treatment are essential in order to conclude that the therapy is effective over and above the non-specific therapeutic effects of compassion, attention, empathic listening and support [37,42]. Therefore, in psychotherapy trials, control groups that offer a comparative amount of supportive personal contact are superior to the use of a wait-list control [37]. Supportive counselling (SC) has often been used as a control condition in psychotherapy trials in adult populations as a control for non-specific relationships effects by offering non-directive, emotional support through the establishment of a therapeutic alliance based on empathic listening [43,44]. There has been only one reported RCT in adolescents with major depressive disorder receiving either CBT, family therapy or SC [45]. CBT was the superior treatment in the shorter-term outcome of symptom reduction, however, the long-term outcomes over two years showed no differences between the groups with regard to recovery, recurrence or level of functioning, with the majority of participants recovering during this time-frame [45]. Both the National Institute for Health and Clinical Excellence (NICE) guidelines [46] and the American Academy for Child and Adolescent Psychiatry practice parameter [47] for treating children and adolescents with depression recommend that those with mild or brief depression initially be offered psychoeducation and supportive counselling. As demonstrated in an RCT of antidepressant medication and routine specialist care with and without CBT (the ADAPT study [24]), a substantial proportion (21%) of child and adolescent participants with moderate to severe major depression responded to a brief intervention that was offered prior to randomisation that included psychoeducation and mental state monitoring, provided within an empathic and supportive framework. As such, SC has face validity as a suitable control condition for young people in the early stages of mild to moderate depressive disorders.

In summary, there is emerging evidence to suggest that PST and exercise interventions may have benefits in reducing symptoms of mental distress, particularly depression, in younger populations, although there is a need for further research that utilises adequate control conditions. It is also of interest to explore the effects of less complex or intense interventions separately and whether there are additional benefits gained from delivering the interventions together.

## Methods/Design

### Study objectives

The primary aims are to evaluate whether simple psychological and exercise treatments are effective for treating sub-threshold or mild to moderate depression and anxiety disorders in young people and, if so, which interventions (or their combination) are most effective for various types or severities of clinical presentations. A secondary aim is to identify possible attributes of those who are likely to respond to less-complex interventions.

It is hypothesised that the active interventions of PST and behavioural exercise will be more effective than the control conditions of supportive counselling and lifestyle psychoeducation in: a) reduction of symptoms of depression and anxiety; b) reduction of substance use; c) improvement in functioning; and d) leading to the remission of depression and anxiety (using either diagnostic thresholds or scale scores, depending on baseline symptomatology). We further hypothesise that: e) the combination of active interventions (that is, PST and behavioural exercise) will lead to a greater reduction in symptoms of depression and anxiety than the combination of control interventions (SC and lifestyle psychoeducation).

### Study design

The study is a factorial RCT (see [48]), as this design allows for multiple interventions to be assessed against appropriate control interventions in the framework of a single trial [48,49] (see Table 1). A factorial design also allows the possibility of considering the effects of both the interventions separately and the effects of delivering the interventions together [48]. It is registered as ACTRN12608000550303 with the Australian New Zealand Clinical Trials Registry and has been approved by the Melbourne Health Human Research and Ethics Committee.

**Table 1:** Factorial RCT study design

		Exercise intervention			
				
		Exercise psychoeducation (control condition)	Behavioural exercise (active condition)		
**Psychological intervention**	Supportive counselling (control condition)	n = 40	n = 40	N = 80	Principal psychological comparison
		
	Problem solving therapy (active condition)	n = 40	N = 40	N = 80	

		N = 80	N = 80		
				
		Principal exercise comparison			

### Setting

*headspace *Western Melbourne (WM) is a youth mental health service that provides assessment and psychological and/or psychiatric treatments to young people aged 12-25 years, in addition to primary health care, vocational and educational assistance and specialist substance use services. These services are delivered in a "one-stop shop", youth-friendly service environment staffed by general practitioners, psychologists, psychiatrists and other allied health professionals, located in the urban Western region of Melbourne, Australia. The *headspace *WM catchment area has a population of 452 762, of which 76 791 are aged 12-24 years. The service is led by a consortium of partners managed by Orygen Youth Health Research Centre.

### Participants

All help-seeking young people aged 15-25 years who present or are referred to *headspace *WM and are on the waiting list are screened for suitability on the basis of study inclusion criteria, with informed consent obtained from all participants. Those under 15 years are excluded, as obtaining parental/guardian consent can be problematic for young people who do not wish to involve their caregivers in seeking help for mental health problems. Inclusion criteria are: depressive/anxious symptoms of more than one week's duration or a decline in functioning over the past month, and no prior formal treatment (e.g., psychological intervention provided by a registered psychologist or pharmacotherapy of at least 4 weeks' duration). Exclusion and withdrawal criteria are: psychotic symptoms and/or specific suicidal plan or intent; already exercising according to Australian government guidelines (i.e., daily moderate to vigorous exercise of 60 minutes duration for under 18 years; four times per week of 30 minutes of moderate to vigorous exercise for those over 18 years [50,51]); organic mental disorder; intellectual disability. Based on power calculations (see below), we aim to recruit 160 participants between April 2009 and July 2011.

### Procedure

After assessing eligibility, a research assistant contacts the young person by telephone to explain the study and offer an appointment for the purpose of obtaining informed consent. Following the provision of informed consent, the research assistant conducts the baseline assessment.

Participants are allocated to their treatment combination prior to the first treatment session with a research psychologist (see randomisation procedure below). At the first treatment session, participants are provided with the relevant psychoeducation materials and resources. During each treatment session, participants' mental state is monitored for the purposes of withdrawal criteria. Treatment continues for the duration of the trial unless the participant is withdrawn due to worsening symptoms that reach threshold level, using the expanded Brief Psychiatric Rating Scale version 4 (ExBPRS; [52]). Threshold levels of severe symptoms and disruption to functioning have been defined as: a score ≥6 on exBPRS Anxiety and Depression subscales or a score of ≥5 on exBPRS Suicidality subscale, maintained for one week; a score ≥5 on exBPRS subscales of hallucinations, suspiciousness, or unusual thought content, and exBPRS score ≥4 on conceptual disorganisation; or the clinical necessity of introducing antidepressant or anxiolytic medication due to distress associated with ongoing symptoms. At the end of the 6 sessions, participants who have not responded or are in need of further clinical intervention are referred to treatment as usual within *headspace *WM, or to another appropriate service.

Structured assessments occur at baseline, mid-point (3 weeks), end-point (6 weeks), and at a 6- and 12-month follow-up.

### Interventions

The interventions are delivered in 6 sessions on a weekly basis by research psychologists and involve a combination of:

a) *Psychological intervention: *problem solving therapy (PST; active condition) or supportive counselling (control condition), AND

b) *Exercise intervention: *behavioural intervention (active condition) or psychoeducation (control condition).

A comprehensive treatment manual for each intervention has been prepared. The PST intervention progressively works through the seven steps of PST, namely (1) identifying the young person's problem/s; (2) selecting one or two key problems; (3) identifying goals; (4) generating solutions; (5) choosing a solution; (6) creating a SMART (specific, measurable, achievable, relevant, time-limited) plan, and (7) reviewing progress/evaluating the plan [33]. The supportive counselling intervention is based on general counselling principles [44] and is guided by the NICE guidelines for young people with mild to moderate depression [46]. The behavioural exercise intervention assists participants in meeting the Australian guidelines for physical activity [50,51] via a process of identifying barriers and strengths for engaging in regular exercise and monitoring physical activities. Participants are provided with psychoeducation on the relationship between exercise and mood/anxiety symptoms, along with exercise diaries and pedometers to record and encourage regular activity.

An important consideration for factorial designs is the feasibility of delivering the interventions together [48]. The interventions were piloted in *headspace *WM with a small group of young help-seekers, and were shown to be acceptable and feasible interventions in this population.

### Outcomes and measures

#### Primary outcome

Depression and anxiety symptoms at the end of treatment and the follow-up points.

#### Primary outcome measures

Beck Depression Inventory-II (BDI-II [53]), Beck Anxiety Inventory (BAI [54]), and Montgomery Åsberg Depression Rating Scale (MADRS [55]).

#### Secondary outcomes

(i) improvement on clinical and self-report measures of symptoms and substance use; (ii) remission (defined as no longer meeting the diagnostic criteria for a disorder if threshold level was reached at baseline, or no longer scoring in the clinical range on scale scores if sub-threshold at baseline); (iii) social and occupational functioning; (iv) exercise frequency and duration; (v) treatment satisfaction; and (vi) any additional treatment post-intervention.

#### Secondary outcome measures

(i) Beck Suicide Inventory, Substance and Choices Scale [56], and the Comprehensive Assessment of At-Risk Mental States (CAARMS [57]); (ii) the Structured Clinical Interview for DSM-IV Axis I disorders (SCID-I; [58]) or the clinical range on scale scores on MADRS (<12), BDI-II (<13) and BAI (<9), depending on diagnostic threshold levels at baseline; (iii) Social and Occupational Functional Scale [59]; (iv) weekly physical activities based on the Active Australia Survey [60]; (v) experience of treatment questionnaire (based on [61]); (vi) additional treatment questionnaire assessing any treatment (psychological, pharmacological, or alternate) between endpoint and the follow-up periods.

### Randomisation and treatment allocation

The randomisation schedule was devised by an independent statistician (AM). Allocation to treatment is concealed as it is carried out by an independent researcher not involved in conducting the interventions or the participant assessments, in accordance with ICH Guideline E9 [62]. Participants are randomised to one of four possible combinations of interventions. The independent researcher contacts the research psychologist after the baseline assessment and prior to the first treatment session to inform them of each participant's treatment allocation. It is not possible to blind the research psychologists and participants to the treatment allocation. All baseline and outcome assessments are, however, conducted by research assistants blinded to treatment allocation.

A stratified randomisation design is used to incorporate characteristics of sex (2 level factor) and symptom severity at baseline (2 level factor: BDI-II cut-off points; ≤28 mild/moderate or ≥29 severe) since any chance imbalances on these variables may prejudice the analyses. Participants are allocated to the treatment groups using randomly permuted blocks within each stratum, to ensure that allocation to treatment groups is approximately equal.

### Statistical analyses

Primary analyses will be conducted on an intent-to-treat basis, including all participants randomised regardless of treatment actually received or withdrawal from the study. Mixed-model repeated measures analyses will be used, as this approach enables the inclusion of participants with missing data, without using inferior techniques such as last observation carried forward [63]. For analyses of binary, ordinal and categorical outcomes, non-linear mixed modelling will be used. Subsidiary analyses will explore participant characteristics that moderate outcome.

### Power analysis

Based on a correlation of 0.7 between baseline and endpoint observations, and allowing for 30% attrition, a sample size of 160 (40 participants in each cell) will maintain power above 75% to detect effects of 0.5 standard deviations change between active and comparison treatments. Beyond the detection of substantial sub-additivity of effects of the interventions, it is recognised that power to differentiate the effectiveness of specific combinations of treatments will not be high. When testing for predictors of effectiveness for individual treatment arms, the study will have 75% power to detect simple relations below r = 0.33 allowing for 30% attrition. The sample size will allow for multivariate analyses with up to 3 predictors, assuming moderate size effects (see [64]).

## Discussion

Whilst preliminary, the emerging evidence-base suggests that simple and less intensive interventions that include a behavioural activation component may be effective in reducing depression and anxiety related symptoms. This paper has outlined a factorial randomised controlled trial that has been specifically designed to test the effectiveness of simple psychological and exercise interventions in young people with sub-threshold or mild-moderate depressive or anxiety disorders, with possible comorbid substance use. The project also aims to identify attributes to determine which clinical presentations are most suitable for simple interventions delivered in a youth mental health service. If effective, the interventions have the potential to prevent the progression of early stages to later and potentially more serious stages of illness and to reduce the likelihood or ongoing problems associated with risk of persistence and recurrence.

## Competing interests

The authors declare that they have no competing interests.

## Authors' contributions

AGP, SEH, AFJ, ARY, PDMcG and RP, conceived of the study, and along with AM and BM, participated in its design. AGP, SEH and RP drafted the manuscript, with input from the remaining authors. All authors have read and approved the final manuscript.
